# Characterization of polydactyly chondrocytes and their use in cartilage engineering

**DOI:** 10.1038/s41598-019-40575-w

**Published:** 2019-03-12

**Authors:** Emma Cavalli, Clara Levinson, Matthias Hertl, Nicolas Broguiere, Oscar Brück, Satu Mustjoki, Anja Gerstenberg, Daniel Weber, Gian Salzmann, Matthias Steinwachs, Gonçalo Barreto, Marcy Zenobi-Wong

**Affiliations:** 1 Tissue Engineering + Biofabrication, Institute for Biomechanics, Swiss Federal Institute of Technology Zürich (ETH Zürich), Otto-Stern-Weg 7, CH-8093 Zürich, Switzerland; 2 Hematology Research Unit Helsinki, Department of Clinical Chemistry and Hematology, University of Helsinki and Helsinki University Hospital Comprehensive Cancer Center, Helsinki, Finland; 3 Division of Hand Surgery, University Children’s Hospital, Steinwiesstrasse 75, 8032 Zürich, Switzerland; 4 Sport Clinic Zürich Hirslanden, Witellikerstrasse 40, 8032 Zürich, Switzerland; 5 Schulthess Clinic, Lengghalde 2, 8008 Zürich, Switzerland

## Abstract

Treating cartilage injuries and degenerations represents an open surgical challenge. The recent advances in cell therapies have raised the need for a potent off-the-shelf cell source. Intra-articular injections of TGF-β transduced polydactyly chondrocytes have been proposed as a chronic osteoarthritis treatment but despite promising results, the use of gene therapy still raises safety concerns. In this study, we characterized infant, polydactyly chondrocytes during *in vitro* expansion and chondrogenic re-differentiation. Polydactyly chondrocytes have a steady proliferative rate and re-differentiate in 3D pellet culture after up to five passages. Additionally, we demonstrated that polydactyly chondrocytes produce cartilage-like matrix in a hyaluronan-based hydrogel, namely transglutaminase cross-linked hyaluronic acid (HA-TG). We utilized the versatility of TG cross-linking to augment the hydrogels with heparin moieties. The heparin chains allowed us to load the scaffolds with TGF-β1, which induced cartilage-like matrix deposition both *in vitro* and *in vivo* in a subcutaneous mouse model. This strategy introduces the possibility to use infant, polydactyly chondrocytes for the clinical treatment of joint diseases.

## Introduction

Globally 1.2 million patients are affected by cartilage degeneration annually, a burden that will increase as population ages^1,2^ and which accounts for one of the leading causes of disabilities worldwide^3^. Additionally, it is estimated that up to 36% of athletes present focal cartilage defects^4^, while up to 69% of adults older than 50 years old show signs of cartilage anomalies in their knees^5^. Articular cartilage has a very limited ability to regenerate due to its low cellularity and lack of vascularization. Consequently, cartilage injuries often lead to the development of post-traumatic osteoarthritis (OA) and frequently require surgical intervention^6^. The limited capability of cartilage to heal has driven the development of cell-based and tissue engineering strategies^7^ such as microfracture, autologous chondrocyte implantation (ACI) and matrix-assisted autologous chondrocyte implantation (MACI). ACI is so far the most effective, clinically approved technique to repair cartilage lesions^8^. However, this technique has major limitations, which include fibrocartilage tissue formation^9,10^, lack of integration of the grafts, the requirement of multiple surgeries and high donor-to-donor variability^11^. These latter drawbacks contribute to more than 20% of non-responders to ACI^12,13^ and justify the need for a next-generation of chondrocyte implantation.

The potential of infant and juvenile cartilage as non-immunogenic, off-the-shelf cell source with stable chondrogenic potential have been extensively investigated and exploited. Infant chondrocytes from deceased donors have been characterized and proposed as a cell source for scaffold-free articular cartilage repair^14,15^ and disc regeneration techniques^16^. Juvenile cells were shown not only to have an enhanced, inherent ability to synthesize cartilage matrix^14^, but also to exhibit immunosuppressive properties^17^. Infant hip chondrocytes from donors with hip dysplasia and Perthes disease in polyglycolic acid (PGA)-fibrin scaffolds were shown to express higher levels of chondrogenic markers and lower levels of undesirable fibroblastic markers compared to adult cells^18^. Clinically, the use of allogeneic, juvenile cartilage has been commercialized since 2007 as DeNovo® NT Natural Tissue Graft from Zimmer. DeNovo® NT is a particulated cartilage implant intended as an early-intervention option for articular cartilage repair and restoration. It was shown to successfully reduce the symptoms associated with cartilage damage, including knee pain, and to improve function and sports activities for at least two years following surgery^19,20^.

**Figure 1:**
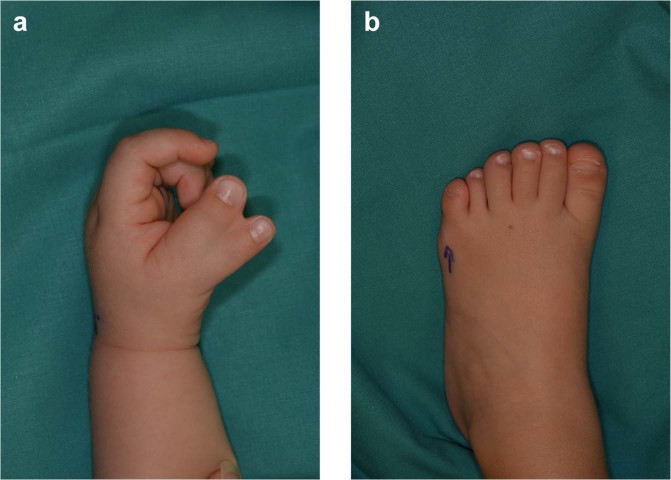
Polydactylous hand (pre-axial polydactyly) (**a**) and polydactylous foot (post-axial polydactyly).

The use of chondrocytes from polydactyly patients overcomes the constraint of the limited availability of healthy deceased donors and tissues from rare disease patients (i.e. Perthes disease). Polydactyly is a congenital malformation that results in the formation of additional fingers or toes (Fig. 1). It has an incidence of 1 in 1000 births on the preaxial side of the hand (thumb duplication) and an incidence of 1 in 3000 births on the postaxial side of the hand and feet (supernumerary little fingers and toes). The incidence highly varies according to ethnicity and is higher in males subjects^21^. The digits often contain fully formed articular joints and are generally removed with corrective surgery at around one year of age^22^. Polydactyly chondrocytes are currently being investigated as an alternative, allogeneic cell source for chondrocyte sheet transplantation^23^. Cell sheet technology has shown promising results already with adult chondrocytes in preclinical studies and in clinical studies with osteoarthritis patients^24^. However, the use of autologous chondrocytes requires a two-step surgical procedure and is associated with high donor-to-donor variability. Additionally, human polydactyly chondrocytes that are retrovirally transduced to express TGF-β1 are currently commercially available in South Korea as INVOSSA (TissueGene-C) and are undergoing phase III clinical trials in the USA. After being proven safe in a range of pre-clinical animal models^25^, INVOSSA chondrocytes have been confirmed safe and efficient in grade III, chronic knee osteoarthritis patients^26,27^. One-year follow-up studies have shown significant improvement in patients treated with INVOSSA^TM^ chondrocytes over the placebo group^28^.

**Figure 2:**
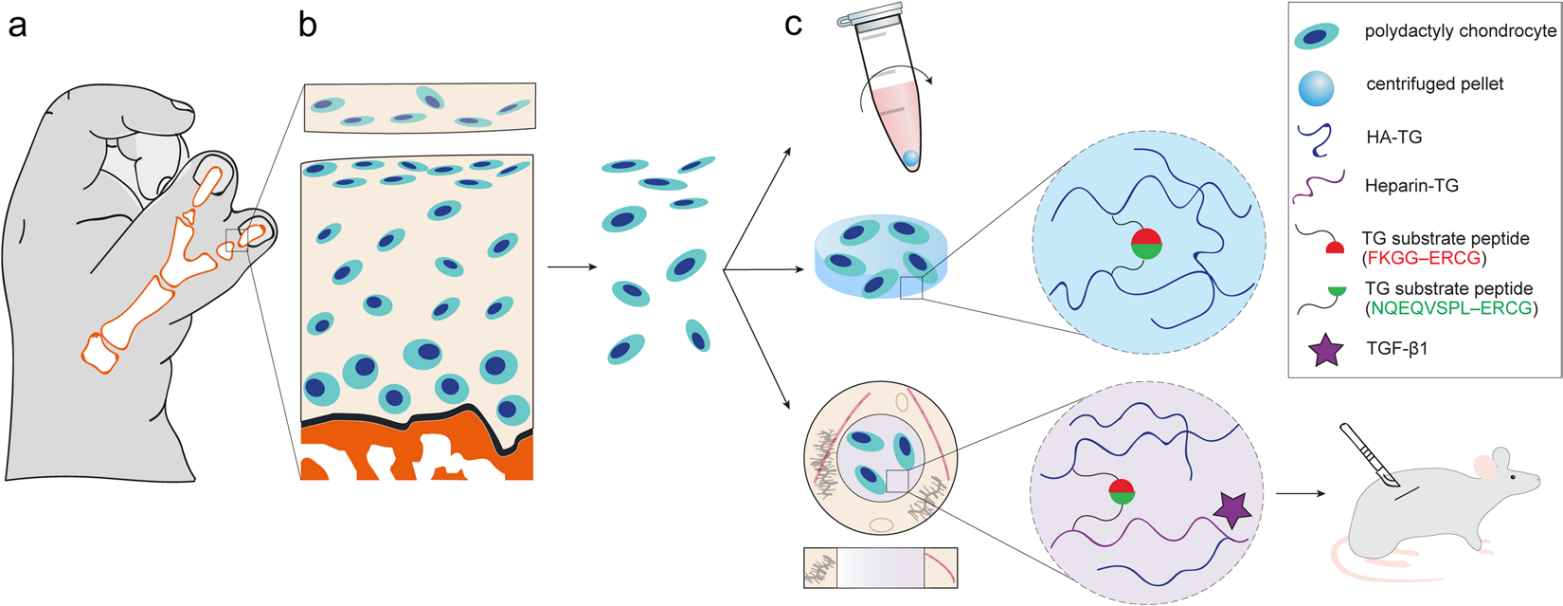
Schematic of the study. Cartilage was harvested from joints of children undergoing surgical correction of polydactyly (**a**) and cells were isolated from the tissue (**b**). Polydactyly chondrocytes were characterized for their de-differentiation profile and for their and re-differentiation ability in 2D and in 3D. 3D studies (**c**) included centrifuged pellet culture and encapsulation in a hyaluronic acid-derived hydrogel, HA-TG. For further *in vivo* studies, the cells were encapsulated in a mix of HA-TG and modified heparin (heparin-TG) in a cartilage explant model for delivery of TGF-β1.

The aim of this study was to evaluate the potential of chondrocytes from polydactyly of children under the age of 2 as a source of cells for articular cartilage repair applications. For comparison, adult chondrocytes were obtained from patients who underwent knee surgical procedures (e.g. MACI). The chondrocytes isolated from articular cartilage of young patients undergoing surgical removal of supernumerary digits are hereby addressed as infant chondrocytes in contrast to the adult chondrocytes. To characterize the properties of the cells the native tissue of polydactyly cartilage was evaluated histologically for cartilage markers and the de-differentiation of polydactyly chondrocytes was characterized up to passage 5. The re-differentiation potential of the cells was evaluated in centrifuged pellets made after sequential passaging. Additionally, we screened for histocompatibility markers and tested the immunosuppressive properties of chondrocytes by performing a T-cell stimulation assay. To evaluate the potential of polydactyly chondrocytes to serve as a cell source for cartilage engineering techniques, we encapsulated the cells into an enzymatically cross-linked hydrogel based on hyaluronic acid modified with tranglutaminase substrate peptides (HA-TG)^29,30^. The ability of the cells to proliferate and produce cartilage-like matrix was studied *in vitro* and in an *ex-vivo* model with TGF-β supplemented medium. Finally, polydactyly chondrocytes were encapsulated in a TGF-β loaded, biomimetic scaffolds and implanted subcutaneously in nude mice (Fig. 2).

## Materials and Methods

### Chemicals

All chemicals were purchased from Sigma-Aldrich unless stated otherwise.

### Cell sources

Human infant chondrocytes (5 male donors and 4 female donors, aged 13 ± 6 months) were isolated from the epiphyseal cartilage in joints removed during corrective surgery for polydactyly.

Human adult chondrocytes (3 female donors and 2 male donors, aged 25 ± 4 years) were isolated from healthy pieces of cartilage obtained during surgical knee operations.

Informed consent was obtained from all adult subjects or from the legal guardians of polydactyly patients. According to the Swiss legal framework, all experimental protocols were approved by the Ethical Committee of the Canton Zürich (Kantonale Ethikkommission, Kanton Zürich) and conducted according to the respective ethical licenses (KEK-ZH 2014-0390 for polydactyly and KEK-ZH 2013-0097 for adult chondrocytes).

### Cell isolation and expansion

Cartilage pieces were minced into 1–3 mm^3^ fragments, washed extensively in PBS with 50 μg/ml gentamicin (Gibco) and digested in collagenase solution (DMEM (Gibco), 0.12% w/v collagenase from Clostridium histolyticum, 10% v/v fetal bovine serum (FBS, Gibco)) overnight with gentle stirring at 30 °C. The resulting cell suspension was passed through a 100 μm cell strainer before collecting the cell pellet by centrifugation (500 rcf, 10 minutes). The cells were plated at 10,000 cells/cm^2^ and expanded in DMEM, 10% v/v FBS, 10 μg/ml gentamicin and 50 μg/ml L-ascorbate-2-phosphate at 37 °C, 5% CO_2_ and 95% humidity.

When reaching 80% confluency, the cells were trypsinized and subcultured to passage 5, plating at 3,000 cells/cm^2^. The growth kinetics was calculated as: n = (log UCY − log l)/log(2) + X, where n is the population doubling at the end of a given subculture, UCY is the cell yield at that point, l is the cell number used as inoculum to begin that subculture, and X is the doubling level of the inoculum used to initiate the subculture^31^. The growth rate was calculated as average population doubling per day.

### Pellet Culture

Cells were suspended in chondrogenic medium consisting of high glucose DMEM supplemented with 10 ng/ml transforming growth factor β3 (TGF-β3, Peprotech), 50 µg/ml L-ascorbate-2-phosphate, 40 µg/ml L-proline, 0.5% penicillin-streptomycin (Gibco) and 1% ITS+ Premix (Corning). Cells were then plated in conical bottom 96-well plates and centrifuged for 5 minutes at 250 rcf to obtain pellets of 250,000 cells each. The cultures were maintained for 3 weeks while the media was replaced 3 times a week.

### Hydrogel preparation and culture

Hyaluronic acid-transglutaminase, or HA-TG, is a novel hydrogel developed by functionalizing high molecular weight hyaluronan with transglutaminase cross-linkable peptides (providing a reactive lysine and glutamine residues respectively). HA-TG was previously shown to be biocompatible, mitogenic and chondroinductive with encapsulated fetal chondroprogenitor cells^30^. Additionally, thanks to its injectability, fast gelation kinetics and adhesion to cartilage, HA-TG was proposed as a novel biomaterial for cartilage engineering applications^30^.

#### HA-TG and heparin-TG synthesis

HA-TG hydrogel precursors, TG/Gln and TG/Lys were synthesized by substituting a hyaluronan backbone (Lifecore Biomedical, 1.01–1.8 MDa) with a reactive glutamine residue (NQEQVSPL-ERCG) and a reactive lysine residue (FKGG-ERCG) respectively as previously described^29,30^. Heparin-TG was synthesized by substituting 15% of the carboxylic acid moieties in the heparin chains with the glutamine-donor peptide. Briefly, 76.2 mg of heparin (heparin ammonium salt from porcine intestinal mucosa) and 4.53 mg of 3,3′-Dithiobis(propanoic dihydrazide) (Frontier Scientific) were solubilized in 7.6 ml of 150 mM 2-(N-morpholino)ethanesulfonic acid (MES) solution. Thereafter 7.32 mg of 1-Ethyl-3-(3-dimethylaminopropyl)carbodiimide (EDC, Fluka) predissolved in a small amount of water was added dropwise and left to react overnight. 26.29 mg of TCEP-HCl (Fluorochem) was added, and the reduction left to proceed overnight. The product was dialyzed against ultrapure water balanced to pH 4.5. The recovered solution was added dropwise into a solution of 190 µl divinyl sulfone (DVS) in 7.6 ml of 300 mM triethanolamine (TEOA) buffer, pH 8.0. The reaction was left to proceed for 8 h at RT and then dialyzed against ultrapure water to yield vinyl sulfone-substituted heparin. The recovered product was functionalized with a substrate peptide that provided a reactive glutamine residue (TG/Gln: NQEQVSPL-ERCG) following the same protocol described in Broguiere *et al*.^29^.

#### Explant model

Stifle joints of 3 to 6 month-old calves were obtained from a local butcher (Metzgerei Angst, Zürich, Switzerland) and dissected to obtain rings with an outer diameter of 8 mm, an inner diameter of 4 mm and a height of 2 to 3 mm. The explants were extensively washed in PBS with 50 µg/ml gentamicin and the inner parts of the rings were digested with 1 U/mL chondroitinase ABC for 15 minutes at 37 °C. The rings were further washed with PBS and kept frozen at −80 °C until use.

#### Collagen scaffolds

Optimaix-3D (1.5 mm in height) is an open porous porcine collagen I/III sponge (containing <30% w/w elastin) produced by a zero-length cross-linking procedure using EDC/NHS chemistry. The scaffolds were kindly provided by Matricel GmbH.

#### Preparation of HA-TG hydrogels

HA-TG precursors, HA/Gln and HA/Lys, were solubilized at 1, 2, or 3% w/v in sterile filtered TBG with calcium (Glucose 100 mM, CaCl_2_ 50 mM, TRIS 50 mM, balanced to pH 7.6). Cells were suspended at the desired concentration in HA-TG precursors. The cross-linking was initiated by adding thrombin (Baxter) and factor XIII (Fibrogammin, CSL Behring) to a final concentration of 12.5 U/ml and 10 U/ml respectively. The gels were quickly cast either in UV-sterilized PDMS cylindrical molds (SYLGARD 184, Corning, diameter = 4 mm, height = 2 mm) adhered to 10 mm coverslips or in the circular hole of cartilage explants (to prevent leakage, the explants were laid on a parafilm-coated surface). The gels were allowed to cross-link for 15 minutes at 37 °C before adding chondrogenic medium. The PDMS molds were then detached from the cover slip and the explants from the parafilm to leave the gels free floating. The cultures were maintained for up to 7 weeks while the media was replaced 3 times a week.

#### Preparation of heparin-TG hydrogels and TGF-β loading

Heparin is a naturally occurring glycosaminoglycan, known to bind certain growth factors, including TGF-β1^32^. In an attempt to more closely mimic the highly sulfated ECM of native cartilage and introduce the possibility of loading growth factors in the hydrogels, heparin was functionalized and incorporated in the HA-TG system. Briefly, the heparin-TG precursor was solubilized in sterile filtered TBG buffer with calcium at a concentration of 0.2% w/v. TGF-β1 (Peprotech) was added to the heparin-TG at a concentration of 1.6 µg/ml and incubated at room temperature for 15 minutes to allow equilibrium binding. 4% w/v HA-TG precursors were solubilized and mixed 1:1 with the heparin-TG precursor. Polydactyly chondrocytes were suspended at 15 million cells/ml in the gel precursor mix and the gelation was triggered as previously described. The final concentrations obtained are 2% w/v HA-TG, 0.1% w/v heparin-TG and 800 ng/ml TGF-β1.

To mechanically support the hydrogels, the addition of a collagen sponge (Optimaix) with a composition similar to that of the clinically used matrix-induced autologous chondrocyte implantation (MACI) scaffolds was investigated. It was hypothesized that the addition of this sponge would improve the HA-TG scaffold system in two ways: on one hand, it reinforces the hydrogel under mechanical compression (Supplementary Fig. 7) and on the other hand it helps retaining the ECM produced by the cells^33^. For this experimental condition, Optimaix scaffolds were punched into cylinders (4 mm in diameter) and placed in the PDMS mold or explant prior to the addition of the HA-TG/cell mixture.

Acellular hydrogels were prepared similarly by solubilizing HA-TG and heparin-TG precursors at the desired concentrations and initiating the gelation as previously described.

### Subcutaneous mouse implantation

Animal studies were performed in accordance with the ethical guidelines of the Health Department of the Veterinary Office of the Canton Zürich (Kanton Zürich Gesutheitsdirektion Veterinäramt, application No. ZH11/2017), complying with the Swiss regulatory framework and following the ETH Zürich Policy on Experimental Animal Research. Female, NU/NU nude mice were obtained from Charles River and operated at 3 months of age. The scaffolds (4 scaffolds/conditions, 2 scaffolds/animal) were prepared and subcutaneously implanted on the same day. Mice were anesthetized with 4.5% isoflurane and Meloxicam (Metacam, 2 mg/kg) was administered via subcutaneous injection before surgery. Eye cream was applied to prevent desiccation of the cornea and the anesthesia was continued with 1.5–3% of isoflurane. Two incisions were made lateral to the dorsal midline at the level of the hip joint and constructs were placed subcutaneously. The incisions were closed with surgical staples, which were removed after 1 week. After 6 weeks, the animals were euthanized by CO_2_ asphyxiation and the explants processed for histology as described below.

### FACS

Human infant chondrocytes were analyzed for the expression of immune-related cell surface markers by flow cytometry using the BD FACSVerse^TM^ (BD Bioscience). Briefly, cells were washed with PBS, centrifuged (300 rcf, 5 min), and the supernatant discarded. Cells were incubated with the surface-marker antibodies CD14, CD3, CD44, CD90 and CD45 (15 min, RT; Characterization panel available in Supplementary Table 1), washed with PBS, and resuspended in PBS-EDTA-BSA. The fluorescence intensity of surface markers in infant chondrocytes was analyzed using FlowJo (v. 10.3.0) and compared with that of mononuclear cells (MNCs) used as positive control.

### T-cell stimulation assay

The immunosuppressive activity of chondrocytes was assessed using allogeneic mononuclear cells (MNCs) with T-cell stimulation assay. Chondrocytes were cultured at various densities (1 × 10^4^, 2 × 10^4^ and 4 × 10^4^ cells/well) with a fixed number of MNCs (2 × 10^5^ cells/well) in RPMI 1640 medium (Lonza) supplemented with 10% fetal bovine serum (FBS), 1% L-glutamine, 1% penicillin and 1% streptomycin in 96-well plates. MNCs with stimulation were incubated with 2.5 μg/ml anti-CD3 (#555329, BD Bioscience), 1 μg/ml anti-CD28 (#340975, BD Bioscience), and 0.5 μg/ml anti-CD49d (#340976, BD Biosciences) for 16 hours (at 37 °C). Unstimulated wells did not include stimulatory antibodies. All wells included GolgiStop (#554724, BD Biosciences). The experiment was conducted in triplicate wells.

After the incubation, cells were stained for surface antibodies as mentioned above (T-cell stimulation panel, available in Supplementary Table 2). For intracellular staining, cells were first treated (15 min, 4 °C) with Fixation/Permeabilization solution (#554714, BD Biosciences) and washed twice with PBS. Anti-interferon gamma (anti-IFNG) was added (30 min, RT), and the cells were washed with PBS and resuspended in PBS-EDTA-BSA before analysis with BD FACSVerse^TM^.

### Histology and immunohistochemistry

Native tissue and explant samples were fixed in 4% formaldehyde in PBS for 4 hours and paraffin embedded. 5 μm sections were cut, deparaffinized with xylene and re-hydrated in decreasingly concentrated ethanol baths before starting the staining protocols. Hydrogel samples (without explants) were fixed in 4% formaldehyde for 1 hour, immersed in 10% w/v sucrose solution in PBS for 2 hours and incubated in 30% w/v sucrose solution overnight. The gels were then embedded in O.C.T (Tissue-Tek Optimum Cutting Temperature Compound, Sysmex) and stored at −80 °C. 5 μm thick sections were cut using a Cryostat (CryoStar NX70, Thermo Scientific). The sections were washed with PBS to remove the OCT before commencing the staining protocols.

Native tissue samples were stained with Alcian blue (pH 2.5) and nuclear fast red, Safranin-O and hematoxylin and eosin (H&E) according to standard protocols.

Collagen 1 and 2 staining were performed after 30 minutes of 1200 U/ml hyaluronidase digestion at 37 °C and 1 hour blocking with 5% normal goat serum (NGS) in PBS with 1:1500 diluted anti-collagen 1 (mouse, Abcam #ab6308 for *in vitro* samples or rabbit, Abcam #ab138492 for *in vivo* samples) and 1:200 diluted rabbit anti-collagen 2 (Rockland 600-401-104). Aggrecan staining was performed after reducing the tissue with 10 mM dithiothreitol (DTT) in 50 mM Tris-HCl and 200 mM NaCl (pH 7.4) for 2 hours at 37 °C and alkylating with 40 mM iodoacetamide in PBS for 2 hours at 37 °C. Sections were then digested with 0.2 U/ml chondroitinase ABC for 40 minutes at 37 °C and blocked for 1 hour at room temperature with 5% NGS before incubating with primary antibody (1:5 dilution, Hybridoma 12/21/1-C-6-s). Lubricin staining was performed after 18 minutes of incubation in trypsin solution (0.05% trypsin, 0.1% CaCl_2_) at 37 °C and 1 hour blocking with 5% NGS in PBS at room temperature, followed by an incubation with 1:250 diluted rabbit anti-lubricin (Abcam #ab28484). All primary antibodies were diluted in 1% w/v NGS (in PBS) and incubated overnight at 4 °C. Alexa Fluor 594 goat anti-mouse IgG (Thermo Fisher Scientific, A11005) and Alexa Fluor 488 goat anti-rabbit (Thermo Fisher Scientific, A11008) secondary antibodies were used at 1:200 dilution in 1% NGS in PBS for 1 hour at RT. Finally, slides were incubated for 15 minutes with the nuclear stain DAPI (Thermo Fisher Scientific) and mounted with VectaMount AQ Mounting Medium (Vector Laboratories).

### PCR

Cell-laden HA-TG hydrogels were collected and frozen. Samples were homogenized using pellet pestles (Thomas Scientific) and RNA was prepared using NucleoSpin miRNA kit (Macherey-Nagel) following manufacturer’s instructions. The RNA concentration was determined with a microplate reader Synergy H1 (BioTek Instruments), then RNA with an absorbance ratio at 260/280 nm between 1.9 and 2.1 was used for polymerase chain reaction (PCR) analysis. The Fast SYBR Green Master Mix (Applied Biosystems) was used to perform the PCR amplification with 150 nM forward and reverse primer. Data was analyzed using the 2^−ΔΔCt^ method and normalized against the house-keeping gene RPL13a^34^. The following primers for human (Microsynth AG) were used: RPL13a (forward (F) 5′ AAGTACCAGGCAGTGACAG; reverse (R) 5′ CCTGTTTCCGTAGCCTCATG), COL1a1 (collagen type 1) (F, 5′ CAGCCGCTTCACCTACAGC; R, 5′ TTTTGTATTCAATCACTGTCGCC), COL2a1 (collagen type 2) (F, 5′ GGAATTCGGTGTGGACATAGG; R, 5′ ACTTGGGTCCTTTGGGTTTG), ACAN (aggrecan) (F, 5′GAATGGGAACCAGCCTATACC; R, 5′ TCTGTACTTTCCTCTGTTGCTG), ChM1 (chondromodulin-1) (F, 5′ TGGAAATAGACGCTGGGAAC; R, 5′ AAAACGAATTCCTGTGATGCC), CHRDL1 (chordin like 1) (F, 5′ GAGATGGAGAACTGTCATGGG R, 5′ GAGAGCGGTGGTAAGAATGTC), MFAP1 (microfibrillar associated protein-1) (F, 5′ CGGCTGGAATGACTACAAGC; R, 5′ TGTCAGGAGGTGCATGTTCT).

### Biochemical assays

Cell-laden HA-TG hydrogels were frozen at −80 °C and digested in papain buffer (250 μg/ml papain from papaya latex in 5 mM L-cysteine HCl, 50 mM sodium phosphate, 2 mM EDTA in water, pH 6.5) overnight at 60 °C with vigorous shaking.

GAG content was determined using the 1,9-DMMB assay. Chondroitin sulfate dilutions were used as standards and absorbance was measured at 595 nm on a microplate reader (Synergy, BioTek Inc.). Total dsDNA was quantified using the Quant-IT PicoGreen kit (Life Technologies) according to manufacturer’s instructions.

### Mechanical testing

#### Compression tests

Pre-hydrated hydrogels were tested under unconfined compression using a TA.XTplus Texture Analyser (Stable Microsystems) with a 500 g load cell and a preload of 0.4 g was applied to ensure that the surfaces of the scaffolds were in direct contact with the cylindrical 20 mm diameter probe. The samples were compressed to a final strain of 10% at a loading rate of 0.01 mm per second. The compressive modulus E was calculated as the slope of the stress–strain curve in the linear range.

#### Push-out tests

Push-out tests were performed on hydrogels in cartilage explants at 0.5 mm/s rate with a 3 mm rod. The bond strength was calculated as the maximum force divided by the integration area in the inner part of the rings.

### Statistical analysis

All experiments were performed with at least 3 different donors, in triplicates and with double technical readings (exception for the proliferation and GAG assays, with only 2 adult donors). All data are reported as mean ± standard deviation of the biological replicates with single data points. For clarity, PCR data are reported as mean ± standard deviation without single data points. SPSS (SPSS for Windows, Rel 20.0.0. Chicago: SPSS Inc.) was used for statistical analysis. Comparison of results was carried out by analysis of variance (ANOVA) using Tukey’s multiple comparison post hoc test for significance. p values of less than 0.05 were considered statistically significant.

## Results

### Extracellular matrix in infant native cartilage

**Figure 3:**
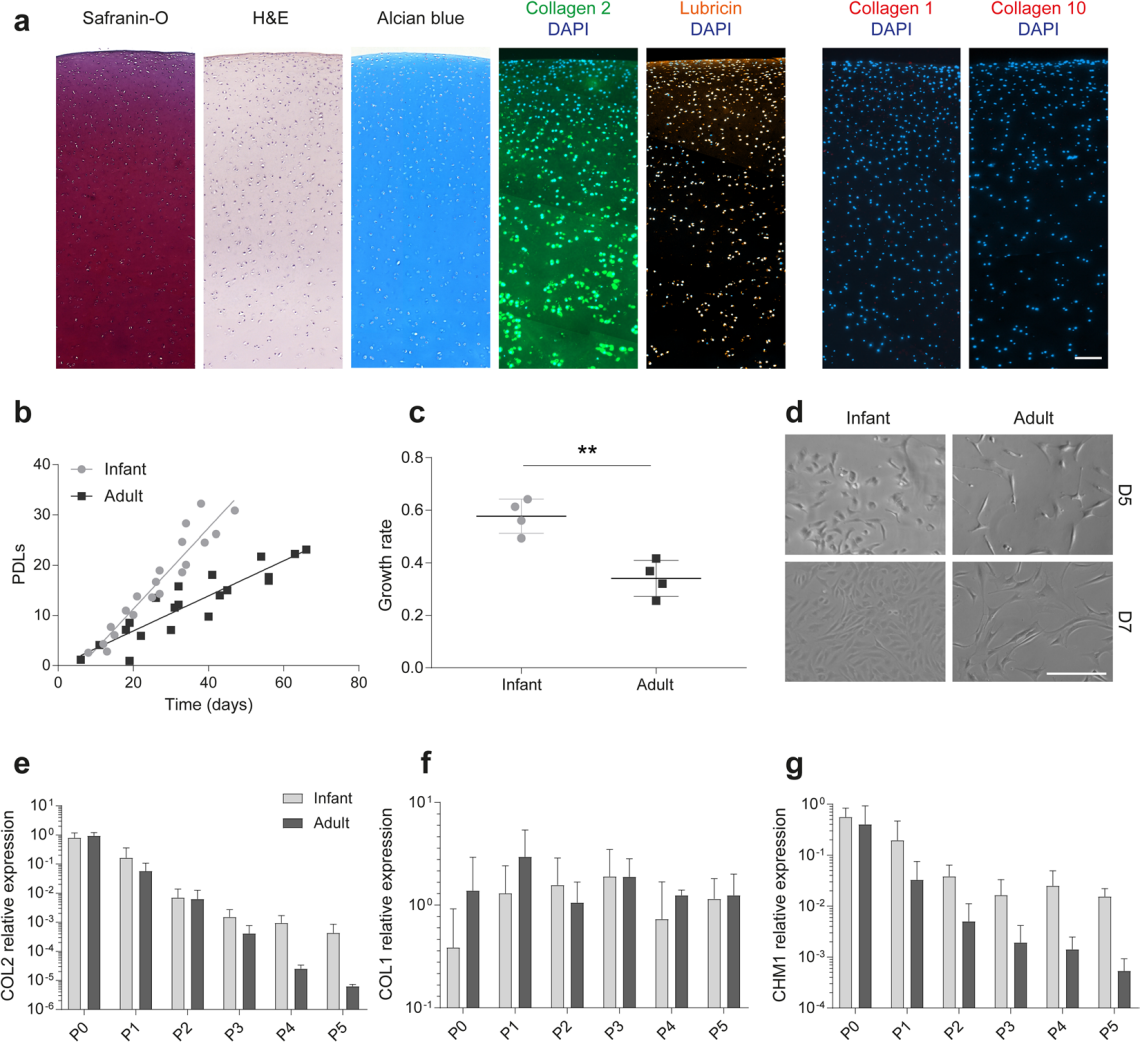
Characterization of infant polydactyly tissue and cells. (**a**) Histological analysis of infant polydactyly cartilage tissue. The images are oriented to present the cartilage surface on the top. Scale bar: 100 µm. (**b**) Growth kinetics of infant and adult chondrocytes expressed as population doubling levels (PDLs) over time. (**c**) Growth rate averages for infant chondrocytes compared to adult chondrocytes, calculated as population doublings per day, **p < 0.01. (**d**) Morphology of infant and adult chondrocytes after 5 and 7 days of 2D culture. Scale bar: 100 µm. (**e**–**g**) Collagen 2 (COL2), collagen 1 (COL1) and chondrododulin-1(CHM1) expression after sequential passaging. Gene expression was normalized against the reference gene RPL13a with one infant chondrocyte donor at passage 0 chosen as the calibrator sample.

Cartilage tissue from joints of surgically resected digits was carefully dissected and histologically analyzed for cartilage-specific markers. Although only the cartilage from the superficial part of the joint (about 1 mm) was used for cell isolation, the whole tissue analyzed exhibited a homogeneous and intense Safranin-O and Alcian blue staining (Fig. 3a) indicating abundant amounts of proteoglycans. Collagen type 2 was present until the deep layer with particularly high intensity in the pericellular zones. Lubricin was expressed mostly in the superficial layer, but also by cells in deeper regions. Lubricin, or proteoglycan 4, is a protein expressed by cells in the superficial cartilage layer to provide boundary lubrication of the tissue but it can be expressed by progenitor cells in deeper layer^35^. No collagen type 1 nor type 10 was found in the cartilage tissue within at least the first 2 mm from the surface.

### Kinetics and de-differentiation profiles in expansion

Infant polydactyly chondrocytes showed a stable and high proliferation capacity during 2D expansion on tissue culture plastic up to passage 5 (Fig. 3b). On the contrary, adult articular chondrocytes displayed a significantly lower (p < 0.01) growth rate (population doubling per day) (Fig. 3b,c) and a more elongated morphology (Fig. 3d) during expansion. The results are in line with what was previously reported in the literature^36^. Additionally, the fast and steady proliferation rate supports the potential of polydactyly chondrocytes as an allogeneic cell source for cartilage repair techniques. Indeed, this would allow to obtain the high number of cells necessary to constitute a master cell bank.

Gene expression analysis of passaged chondrocytes showed that both adult and infant chondrocytes de-differentiate during *in vitro* culturing and expansion. Collagen 2 expression, a typical chondrocyte marker, decreased up to 100,000 fold for adult chondrocytes and up to 1,000 fold for polydactyly chondrocytes when the cells where expanded to passage 5. Similarly, Chondromodulin-1, a strong anti-angiogenic factor and ossification inhibitor^37^, was downregulated in both cell types during *in vitro* expansion but reached a plateau at passage 3 in infant chondrocytes. Collagen 1, a fibroblastic marker, did not show a strong upregulation in either cell type.

We also investigated the gene expression of two recently identified “juvenile chondrocyte-specific factors”, chordin-like 1 (CHRDL1) and microfibrillar-associated protein (MFAP4)^38^. Chordin-like 1 is a bone morphogenic protein (BMP) antagonist known to be involved in connective tissue development^39^, while microfibrillar-associated protein 4 is an extracellular matrix (ECM) protein involved in collagen binding^40^. Both genes were upregulated in infant chondrocytes compared to adult chondrocytes. The trend could be observed both for freshly isolated cells and for extensively passaged cells (Supplementary Fig 1). Collectively, these results confirm the previous studies supporting the age-related involvement of the genes and show that these genes are not only expressed by freshly-isolated infant chondrocytes but also by passaged cells.

### Immunophenotyping of infant chondrocytes

**Figure 4:**
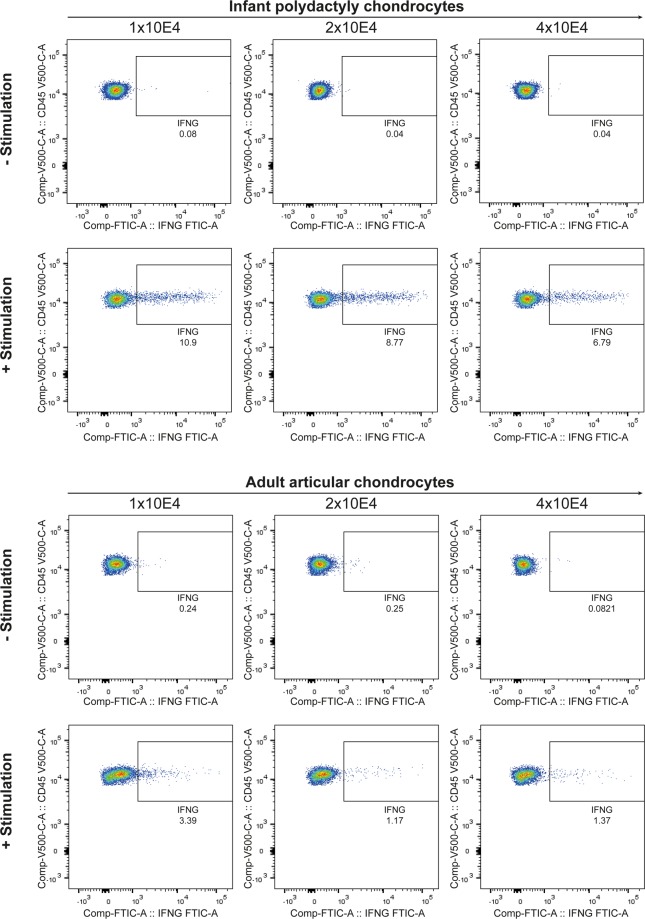
CD4^+^ T-cell stimulation assay. Polydactyly and adult chondrocytes were co-cultured at different seeding numbers with T lymphocytes. Chondrocytes do not trigger CD4^+^ T lymphocytes reaction and, when T lymphocytes were activated, chondrocytes demonstrated an inhibitory behavior as observed in the decreased secretion of interferon gamma (IFNG). CD4+ T lymphocytes, shown on y-axis as CD45-V500, were gated using the strategy available in Supplementary Fig. 3.

Due to its avascular nature and lack of lymphatics, cartilage is considered immunoprivileged and allograft transplantation can be performed even in absence of immunosuppressive therapy^41,42^. Additionally, juvenile chondrocytes were shown to inhibit T cell proliferation via cell surface B7 co-stimulatory molecules and through high expression of chondromodulin-1 and of indoleamine 2,3-dioxygenase (IDO)^17^. Polydactyly chondrocytes were also observed not to induce a T cell response thanks to the absence of major histocompatibility complex (MHC) class II and T cell co-stimulatory molecules^43^. However, they express T cell co-inhibitory molecule PD-L2 and suppress T cell proliferation from a MHC-mismatched donor^43^. In this study we observed that polydactyly chondrocytes do not express the surface immune-related markers, CD45, CD14 and CD3 (Supplementary Fig. 2) but express the chondrocytic markers CD44 and CD90. We also performed a T-cell stimulation assay where polydactyly and adult chondrocytes were co-cultured at different seeding numbers with a fixed number of T lymphocytes. We observed that polydactyly, but also adult chondrocytes, do not trigger T lymphocytes reaction, in either CD4^+^ or CD8^+^ T lymphocytes. Moreover, when T lymphocytes were activated, chondrocytes demonstrated a concentration-dependent inhibitory behavior as observed in the decreased secretion of IFNG in both CD4^+^ or CD8^+^ T lymphocytes (Fig. 4 and Supplementary Fig. 4).

### Chondrogenic re-differentiation of infant chondrocytes in pellet culture

**Figure 5:**
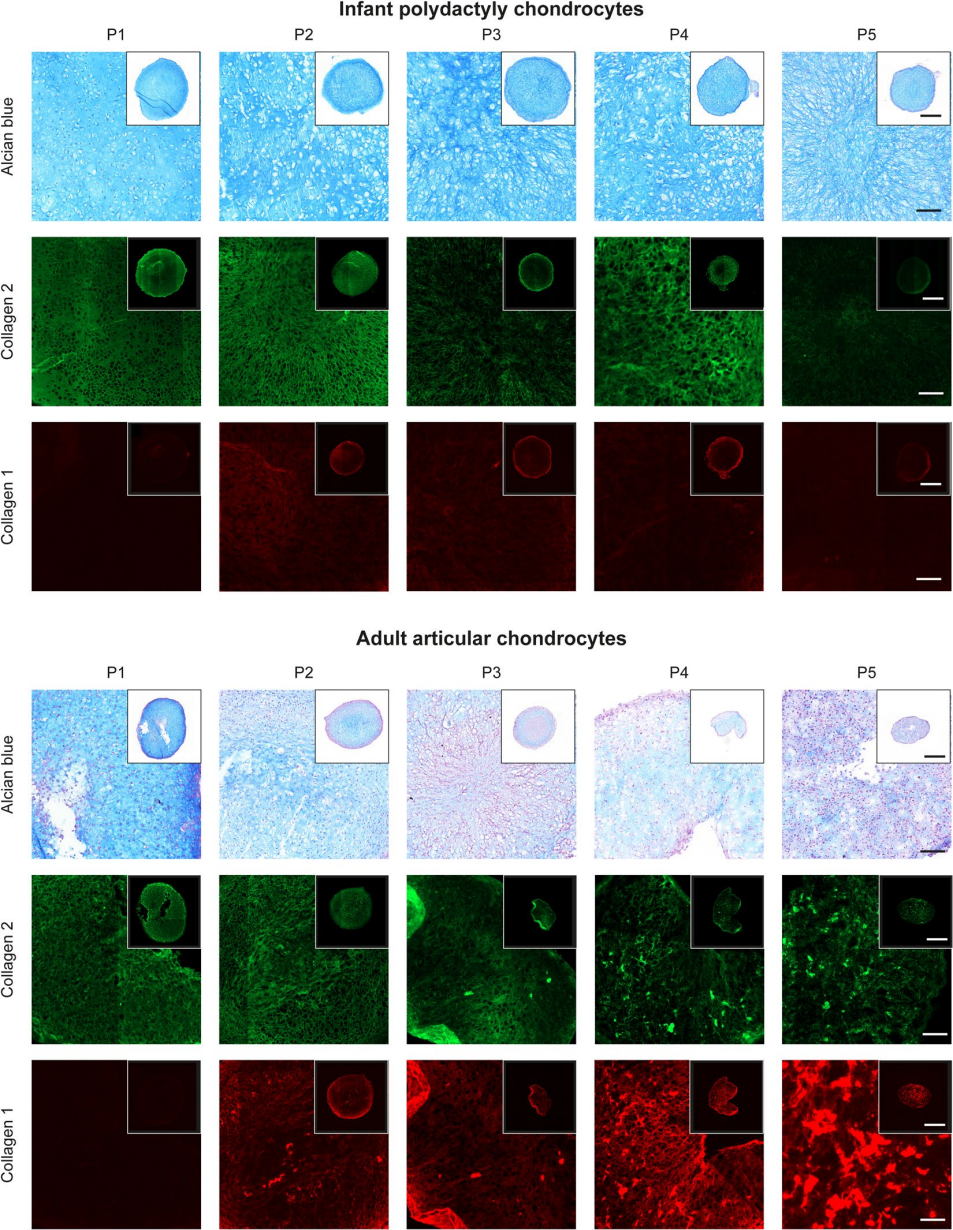
Chondrogenic re-differentiation of infant polydactyly chondrocytes and adult articular chondrocytes in centrifuged pellet culture. Alcian blue, collagen 2 and collagen 1 staining of centrifuged pellets made with 250,000 cells/pellet after 3 weeks of *in vitro* culture in chondrogenic media. Scale bar: 100 µm, scale bar insert: 500 µm.

Centrifuged pellet culture is a widespread assay for recapitulating embryonic condensation and inducing chondrogenic differentiation of mesenchymal stem cells^44^. High-density cultures are known to be a model for re-differentiation of de-differentiated, passaged chondrocytes^45^. Despite the downregulation of chondrocyte-specific markers during *in vitro* passaging infant polydactyly chondrocytes were able to deposit significant amount of cartilaginous ECM after 3 weeks of centrifuged pellet culture in chondrogenic media. Cells exhibited a homogeneous distribution of glycosaminoglycans (GAGs) as shown by the Alcian blue staining until passage 5 (Fig. 5 and Supplementary Fig. 5). Additionally, collagen 2 was highly and homogeneously expressed until passage 4 without a strong upregulation of collagen 1. On the other hand, adult articular chondrocytes produced smaller pellets with a high deposition of GAGs only until passage 1 and a pericellular expression of collagen 2 after passage 3. Collagen 1 was produced incrementally from passage 2 to 5. These results demonstrate the ability of infant polydactyly chondrocytes to re-differentiate towards the chondrogenic lineage even after extensive passaging. This ability is highly advantageous compared to adult chondrocytes as it allows the creation of a cell bank of off-the-shelf chondrocytes.

### Chondrogenic re-differentiation of infant chondrocytes in 3D hydrogels

**Figure 6:**
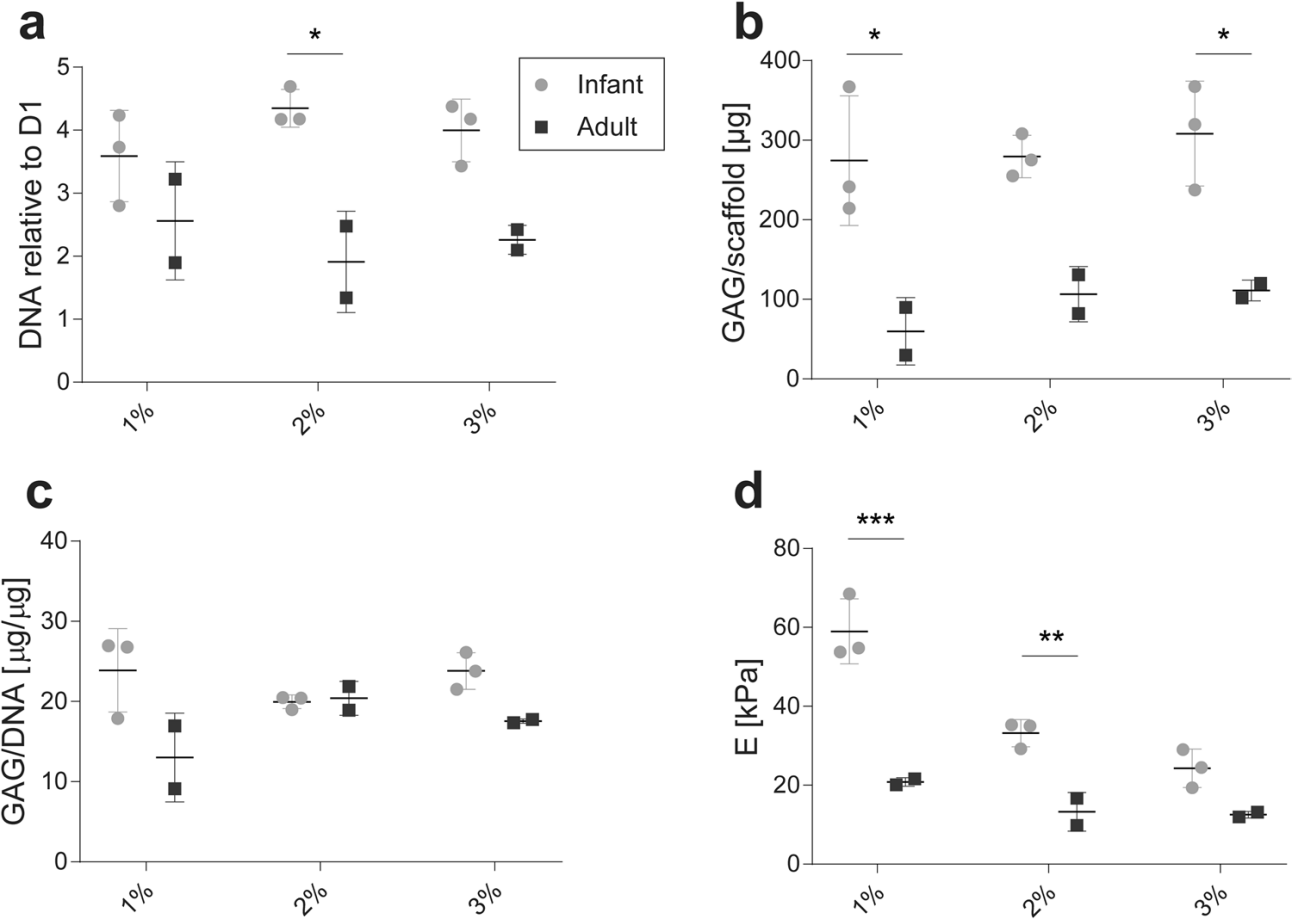
Proliferation and GAG production of P3 infant polydactyly chondrocytes and P3 adult articular chondrocytes in HA-TG hydrogels. Cells were encapsulated in HA-TG at different concentrations (1, 2 and 3% w/v) and cultured for 3 weeks *in vitro* in chondrogenic media. (**a**) DNA fold increase to day 1 (D1). (**b**) GAG amount per scaffold. (**c**) GAG normalized to DNA content per scaffold. (**d**) Compressive modulus E. *p < 0.05, **p < 0.01, ***p < 0.001.

We evaluated the ability of infant and adult chondrocytes to proliferate and produce cartilaginous ECM in HA-TG. Infant polydactyly and adult articular chondrocytes were encapsulated at 15 million cells/ml in 1, 2 and 3% w/v HA-TG and cultured for 3 weeks *in vitro*. Both cell types proliferated during the culture, with polydactyly chondrocytes having consistently higher DNA content in all of the conditions tested (Fig. 6a). Independently of the HA-TG concentration, both cell types secreted GAGs in the hydrogel (Fig. 6b). When the GAG amount was normalized to the DNA in the scaffolds, no significant difference was observed between the two cell types for any of the HA-TG concentrations (Fig. 6c), indicating that the conditions induce similar GAG deposition per cell. As a consequence of the ECM deposition by the encapsulated cells, hydrogels increased their compressive moduli over the 3 weeks of culture (Supplementary Fig. 6 and Fig. 6d), going from 1.3 ± 0.3 kPa for the initial 1% gels to 58.0 ± 5.5 kPa and 20.9 ± 2.8 kPa for infant and adult chondrocytes respectively, from 4.6 ± 1.4 for the 2% gels to 33.5 ± 10.2 and 13.3 ± 4.5, and from 7.0 ± 3.5 kPa for the 3% gels to 24.3 ± 6.7 and 12.54 ± 2.5 kPa.

**Figure 7:**
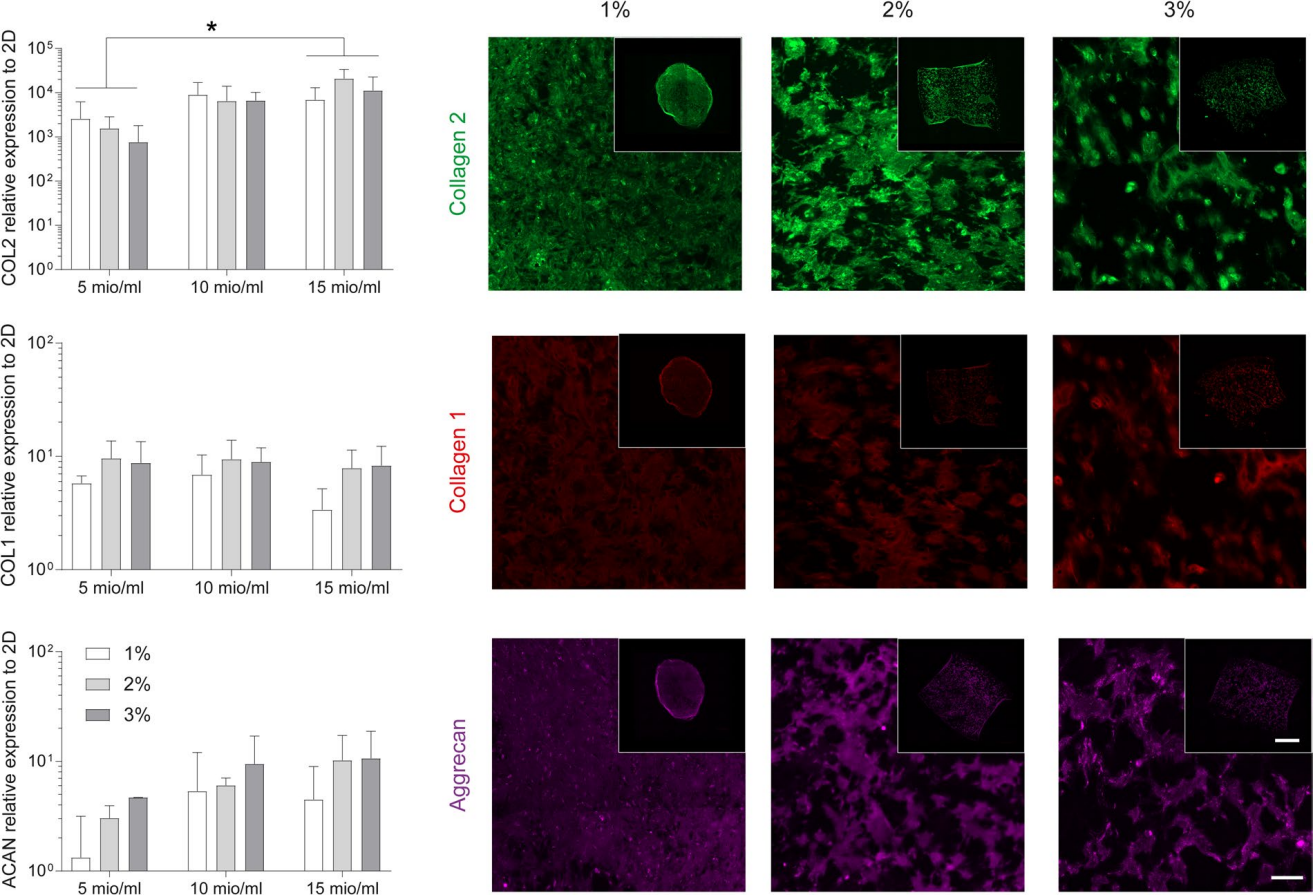
Gene expression and matrix protein deposition by infant polydactyly chondrocytes encapsulated in 1, 2 and 3% w/v HA-TG and cultured for 3 weeks *in vitro* in chondrogenic media. Gene expression of collagen 2, collagen 1 and aggrecan was evaluated for infant chondrocytes encapsulated at 5, 10 and 15 million cells/ml, while protein deposition is shown for the 15 million cells/ml concentration only. Scale bar: 100 µm, insert: 1 mm. *p < 0.05.

The results indicate that HA-TG supports proliferation and matrix deposition of encapsulated chondrocytes, particularly of infant cells. Therefore, we tested whether polydactyly infant chondrocytes would re-differentiate and produce cartilage-specific proteins in HA-TG at different cell seeding densities (Fig. 7). 5, 10 and 15 million cells/ml were encapsulated in 1, 2 and 3% w/v HA-TG hydrogels and cultured for 3 weeks in chondrogenic media *in vitro*. In all of the conditions, infant chondrocytes highly upregulated (1,000 to 10,000 fold) collagen 2 expression compared to passage 3, 2D controls. Similarly, collagen 1 and aggrecan were upregulated up to 10 fold. The highest cell seeding density condition (i.e. 15 million cells/ml) consistently led to the upregulation of cartilage-specific markers (collagen 2 and aggrecan) and to lower expression of the fibroblastic marker collagen 1.

Histological analysis of the 15 million cells/ml hydrogels revealed abundant protein deposition (Fig. 7). 1% gels induced a homogeneous production of collagen 2, 1 and aggrecan that filled the whole hydrogels. However, they tended to shrink in size during the culturing time. ECM deposition was more concentrated around cell clusters in the stiffer gels and the higher concentration of hydrogel led to poorer collagen 2 deposition. Therefore, for further studies, the intermediate stiffness condition (2%) was chosen.

### *Ex vivo* model and growth factor loading

Infant chondrocyte-seeded HA-TG, with and without additional heparin-TG and Optimaix scaffolds, were cross-linked *in situ* in bovine cartilage explants. The constructs were then cultured for up to 6 weeks *in vitro* in presence or absence of TGF-β1. The growth factor was either mixed with the hydrogel precursors before cross-linking (this condition is indicated as “TGF-loaded”) or added to the media (indicated as “soluble TGF”).

**Figure 8:**
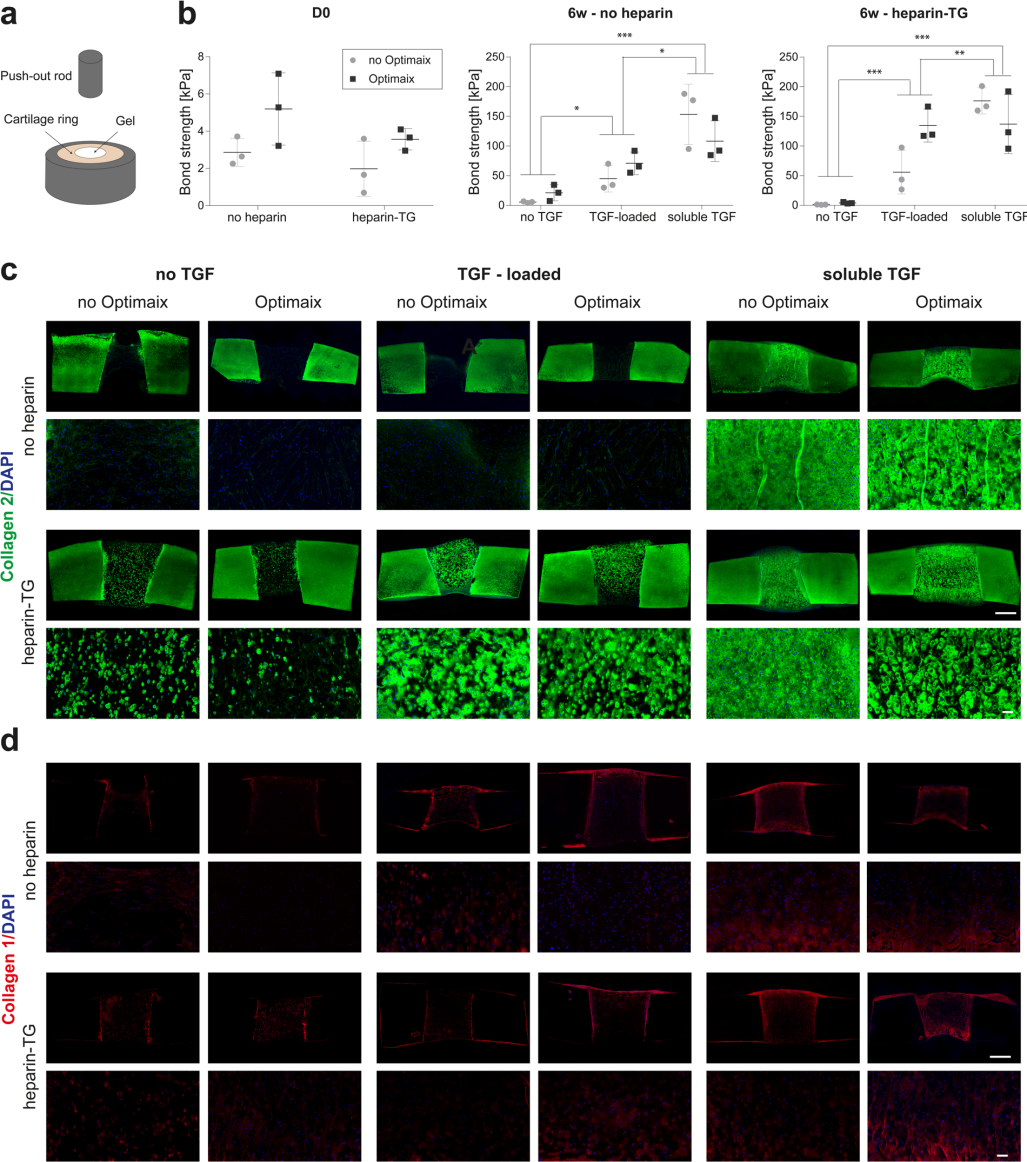
Explant model *in vitro*. Constructs were either cultured in TGF-free media (“no TGF”) or loaded with TGF and cultured in absence of additional factors (“TGF-loaded”) or cultured in chondrogenic media containing TGF (“soluble TGF” condition). (**a**) Schematic of the push-out test. (**b**) Bond strength of the constructs at D0 and after 6 weeks of *in vitro* culture. *p < 0.05, **p < 0.01, ***p < 0.001. (**c**,**d**) Collagen 2 and collagen 1 staining of the constructs with DAPI counterstaining. The top picture depicts the whole construct, while the bottom one depicts a close up of the middle part of the hydrogel. Scale bar: 1 mm for whole construct and 100 µm for close-up.

Neither the addition of heparin-TG nor the addition of Optimaix affected the high adhesive properties of HA-TG to native cartilage as indicated by push-out test done on cartilage explants shortly after cross-linking (Fig. 8). After 6 weeks of culture, scaffolds cultured in presence of TGF-β1 (loaded and soluble) exhibited higher bond strength to cartilage, likely due to matrix deposition at the interface between the cartilage and the hydrogel. As reflected by the push-out tests, infant chondrocytes cultured in HA-TG produced limited amount of collagens in absence of TGF-β1. Interestingly, the addition of heparin to the hydrogel induced some spontaneous collagen 2 production without affecting either collagen 1 production or cell viability.

As expected, mixing TGF-β in HA-TG did not induce visible collagen production as the growth factor is expected to quickly diffuse out and get washed in media changes. On the other hand, when heparin–TG was blended with HA-TG, collagen 2 deposition was achieved, reflecting growth factor retention. The highest collagen 1 and 2 staining intensities were obtained when TGF-β was delivered to the cells constantly by media supplementation.

The addition of the Optimaix scaffold did not seem to have a visible effect on the constructs in terms of bond strength, matrix deposition and cell viability at 6 weeks of culture.

### Subcutaneous *in vivo* study

Finally, we wanted to test the ability of polydactyly chondrocytes to produce cartilage-like matrix in HA-TG with heparin-TG in an ectopic mouse model. Cartilage explant constructs were prepared by cross-linking 2% HA-TG with 0.1% heparin-TG in cartilage explants. Some of the constructs were pre-loaded with 800 ng/ml of TGF-β1 (20 ng/construct) by incubating the hydrogel precursors with the growth factor for 10 minutes before initiating the gelation. Of these TGF-β-loaded constructs, some were further reinforced with Optimaix scaffolds. The constructs were implanted subcutaneously in nude animals within 4 hours after casting and explanted after 6 weeks.

**Figure 9:**
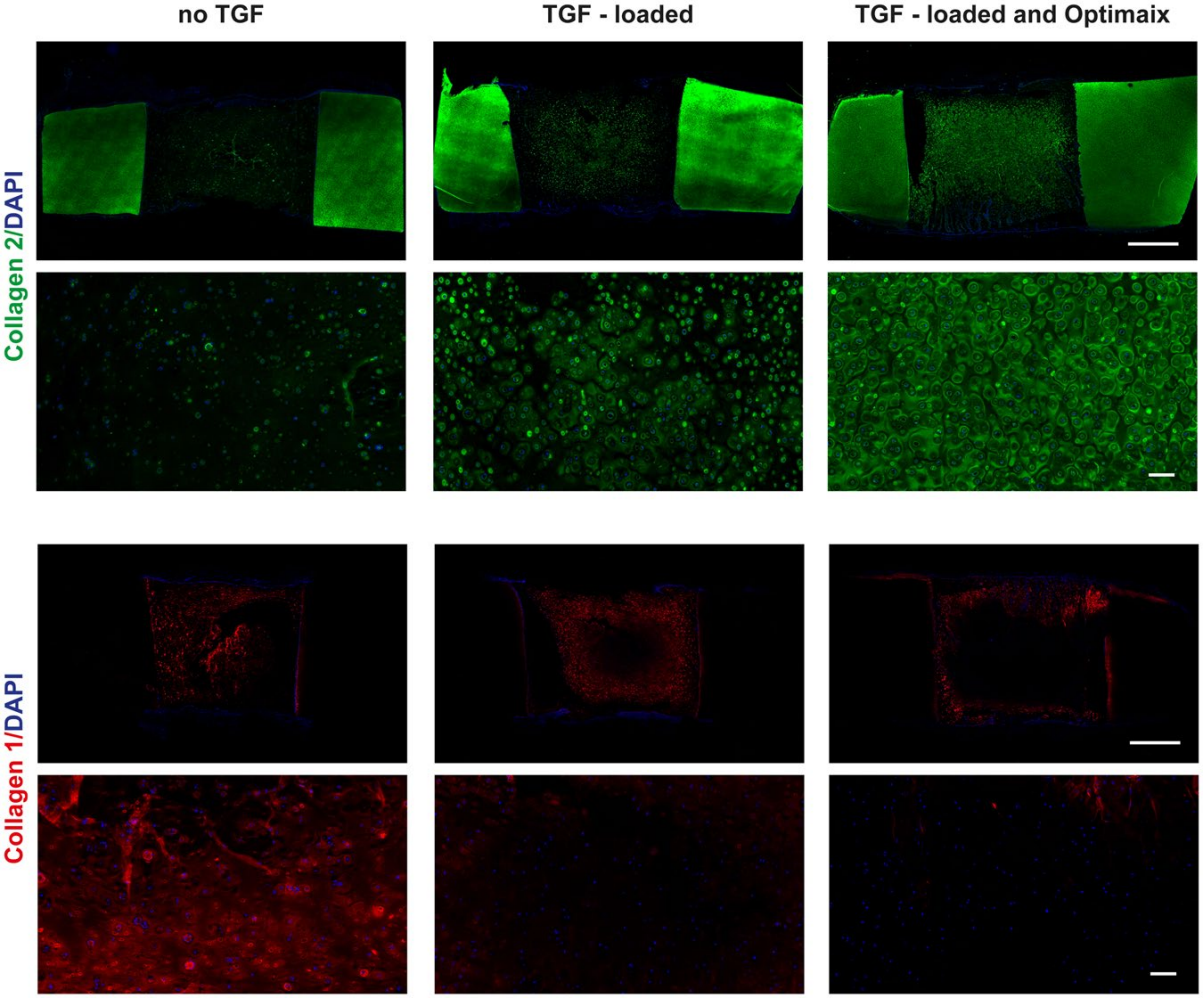
Chondrogenesis *in vivo*. HA-TG scaffolds with the addition of heparin-TG were implanted subcutaneously in nude mice and explanted after 6 weeks. Collagen 2 and collagen 1 staining of the constructs with DAPI counterstaining of constructs without growth factor loading (no TGF), loaded with TGF (TGF-loaded) and TGF-loaded with the addition of Optimaix scaffolds (TGF- loaded and Optimaix).

Collagen 2 analysis (Fig. 9) of un-loaded constructs only showed sparse, pericellular staining, indicating that this ECM protein was either not synthesized or not retained in the hydrogels. Contrarily, abundant collagen 1 could be observed. Growth-factor loading did not affect cell viability and successfully induced collagen 2 production while reducing collagen 1 deposition, especially in the inner part of the gels. The further addition of the Optimaix scaffold facilitated matrix retention and resulted in a homogeneous deposition of collagen 2 and only minimal collagen 1, without visibly affecting cell viability.

## Discussion

The recent increase in popularity of cell-based strategies for cartilage repair application has generated an urgent need for a potent and safe off-the-shelf cell source. The availability of autologous chondrocytes is limited and requires costly and time-consuming expansion. Additionally, the need for a biopsy introduces donor site morbidity and, in the case of articular chondrocytes, often leads to the production of fibrocartilage tissue^46^. Mesenchymal stem cells (MSCs) have been used in several clinical applications, however, their presence and chondrogenic differentiation capability are highly donor-to-donor dependent^47,48^.

Recently, allogeneic cell sources emerged as possible clinical alternatives to autologous approaches, with several advantages. First, patients do not need to undergo an additional surgery, making the surgical procedure faster and logistically simpler. Second, there is no donor site morbidity and no lagtime required for *ex vivo* expansion. Treating the lesion immediately after injury helps to prevent ulceration of the tissue and reduces the risk of post-traumatic OA^49^. Finally, outcome variability can be reduced by using a single donor or a pool of established donors to treat several patients. For these reasons, allogeneic cell-based treatments are already at the clinical trial stage in several countries for chronic osteoarthritis indications. It was demonstrated that allogeneic bone marrow MSC transplantation is not only feasible and safe, but it can also improve pain, quality of life and cartilage quality compared to a biomaterial alone^50^. Invossa TissueGene C, an allogeneic cell and gene therapy based on TGF-β1 transfected polydactyly chondrocytes, is currently commercially available in South Korea and undergoing phase III clinical trials in the USA for severe osteoarthritis indications. Cells are irradiated to stop their reproduction circle and delivered via one intra-articular injection without a carrier into patients’ knees. Randomized, placebo-controlled trials demonstrated feasibility, safety and efficacy in improving pain and function after up to 24 months^28^.

Due to the recent clinical trials and the promising results obtained with allogeneic and infant chondrocytes, there is an increasing interest in the use of polydactyly chondrocytes as a cell source for cartilage repair. Nevertheless, polydactyly chondrocytes have been characterized and used only by a handful of research centers. In this study, we isolated polydactyly chondrocytes from cartilage tissue obtained by surgical resection of supernumerary digits and we characterized their de-differentiation and re-differentiation profiles *in vitro* and *in vivo*. We could demonstrate that polydactyly chondrocytes can be isolated from their native tissue with an easy and fast protocol by enzymatic digestion. Despite the limited size of the tissue, cellularity in young cartilage is higher than in mature, adult tissue^51^, leading to a relatively high cell yield compared to adult tissue. Additionally, with an incidence of up to 1 in 500 births, polydactyly is a common malformation making polydactyly chondrocytes an available source of cells.

We could show that polydactyly chondrocytes can be expanded *in vitro* for up to 5 passages maintaining a fast and steady proliferative rate, an important characteristic for an off-the-shelf cell source. The higher proliferation rate of infant chondrocytes is consistent with the reduction in telomerase length and activity associated with chondrocyte senescence. It was shown that telomerase activity in articular chondrocytes is lost after puberty, leading to a reduction in cell proliferation and life span^52^. We investigated the de-differentiation profile of the cells under *in vitro* expansion, and we could confirm that polydactyly chondrocytes are not only non-immunogenic, but also immune-suppressive. Even though polydactyly chondrocytes tend to lose their chondrogenic phenotype during expansion (i.e. downregulation of collagen 2 expression), they are highly responsive to chondrogenic induction. Indeed, we could show that 3D culture in centrifuged pellets of expanded polydactyly chondrocytes is able to induce chondrogenic re-differentiation and lead to cartilaginous matrix production. The chondrogenic potential of chondrocytes have been shown to decline dramatically with age and infant chondrocytes were reported to have a higher ability to repair cartilage lesions and higher response to growth factors^15,53^. These characteristics are associated with a different gene expression profile in infant chondrocytes which include a higher expression of juvenile-specific genes (e.g. chordin-like 1 and microfibrillar-associated protein 4) and an enrichment of stem cell-like factors^38^.

Finally, we could show that passaged infant polydactyly chondrocytes can be encapsulated and re-differentiated in 3D hydrogels both *in vitro* and *in vivo* in a subcutaneous mouse model. Previous studies using transduced and non-transduced polydactyly chondrocytes focused on scaffold-free approaches^28,54^. Invossa TissueGene consists of intra-articular injections of TGF-β-transduced polydactyly chondrocytes, while Nasu *et al*. have shown that subcutaneous injections of non-transduced polydactyly chondrocytes in immunodeficient mice leads to chondrogenesis and osteogenesis via endochondral ossification. Clinically, the use of a biomaterial for cartilage repair applications has been shown to be beneficial in comparison to scaffold-free approaches. Biomaterials can indeed retain the implanted cells in place preventing their leakage from the lesion site and, at the same time, support cell survival and ECM production^55^. Smeriglio *et al*. showed that poly(ethylene glycol) (PEG) and chondroitin sulfate-based hydrogels supports the chondrogenic phenotype of articular infant chondrocytes^56^. To our knowledge, our study is the first attempt to re-differentiate polydactyly chondrocytes in a 3D biomimetic hydrogel. We chose to use HA-TG because it is based on the cartilaginous GAG hyaluronic acid and can be cross-linked using cell-friendly chemistry. Additionally, it is highly versatile and can be co-cross-linked with transglutaminase-modified heparin. The addition of heparin and the consequent sulfation, makes the material more biomimetic and cell-instructive. Importantly, it also introduces the possibility of loading the scaffolds with heparin-binding proteins. This means that TGF-β1 can be incorporated in the scaffolds and released to the cells. It is known that polydactyly chondrocytes require a constant supply of chondrogenic factors to maintain a chondrogenic phenotype after being passaged in monolayer culture^57^. Growth factor loading in the hydrogel represents an interesting alternative to viral transfection of chondrocytes. In fact, despite the recent advances in gene therapy, the use of retroviral vectors still raises safety concerns^58,59^.

Further studies will be necessary to confirm whether our hydrogel system would induce and maintain chondrogenic differentiation of polydactyly chondrocytes upon implantation in a cartilage-inducing/maintaining environment, such as an articular cartilage defect. Tests in a chondral defect in a large animal model such as a sheep or goat will be informative as to the clinical translational potential of the cells and material combination.

## Conclusions

In conclusion, we could demonstrate that due to their high proliferative rate, immunosuppressive properties and high responsiveness to chondrogenic induction, polydactyly infant chondrocytes represent a promising cell source for allogeneic cell-based therapies for cartilage repair. In addition, infant polydactyly chondrocytes can proliferate and produce cartilage-like matrix in 3D biomimetic hydrogels in the presence of TGF-β. The versatility of our hyaluronic acid-based hydrogel allows the release of the chondrogenic factor TGF-β and the reinforcement with collagen sponges, opening the opportunities to use polydactyly chondrocytes for focal cartilage defect treatment and not only for osteoarthritis. We could demonstrate that polydactyly chondrocytes are able to produce cartilaginous ECM *in vivo* and in absence of pre-culturing.

## Supplementary information

Supplementary dataset
