# Supplementary material for: Characterization of polydactyly chondrocytes and their use in cartilage engineering
Source: Sci Rep. 2019 Mar 12;9:4275. doi: 10.1038/s41598-019-40575-w (PMC6414529; doi:10.1038/s41598-019-40575-w)
Supplement: Supplementary file 1 — Supplementary dataset [file 41598_2019_40575_MOESM1_ESM.pdf]

Characterization of polydactyly chondrocytes and their use in cartilage engineering

Emma Cavalli, Clara Levinson, Matthias Hertl, Nicolas Broguiere, Oscar Brück, Satu Mustjoki, Anja Gerstenberg, Daniel Weber, Gian Salzmann, Matthias Steinwachs, Gonçalo Barreto, Marcy Zenobi-Wong

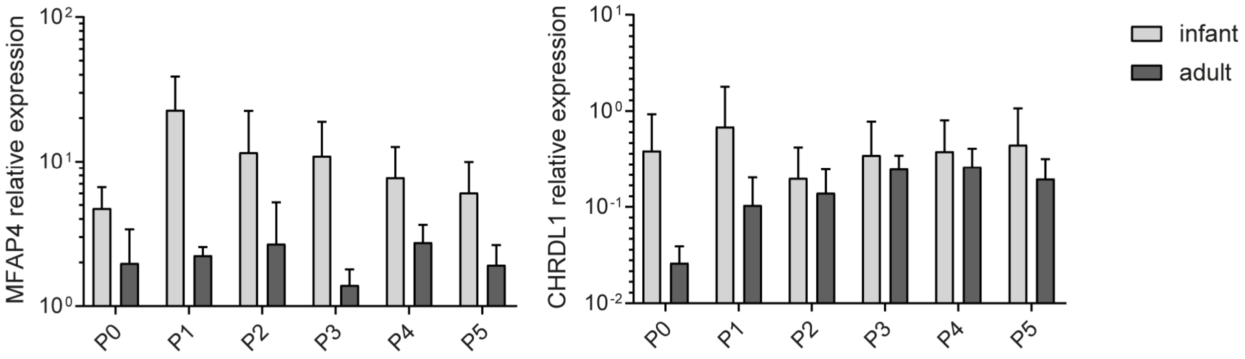

Supplementary Figure 1 Microfibrillar-associated protein 4 and chordin-like-1 expression after sequential passaging. Gene expression was normalized to the reference gene RPL13a with one infant chondrocyte donor at passage 0 chosen as the calibrator sample.

Supplementary Table 1 Markers and respective antibodies used in the FACS characterization panel.

Marker	Fluorochrome	Volume/well (µl)	Catalog number	Company
CD14	PerCP-Cy5.5	5	550787	BD Biosciences
CD3	AF700	1	557943	BD Biosciences
CD44	APC	5	17-0441-81	eBioscience
CD90	PeCy7	5	561558	BD Biosciences
CD45	V500C	3	655873	BD Biosciences

Supplementary Table 2 Markers and respective antibodies used for T-cell stimulation assay.

Marker	Fluorochrome	Volume/well (µl)	Catalog number	Company
CD45	V500C	3	655873	BD Biosciences
CD3	BV421	3	563798	BD Biosciences
CD4	PE	3	555347	BD Biosciences
CD8	Pe-Cy7	1	335822	BD Biosciences
IFNg	FITC	0.6	554700	BD Biosciences
IL-10	PerCP-Cy5.5	4	501418	BioLegend
GrB	Alexafluor700	0.9	561016	BD Biosciences

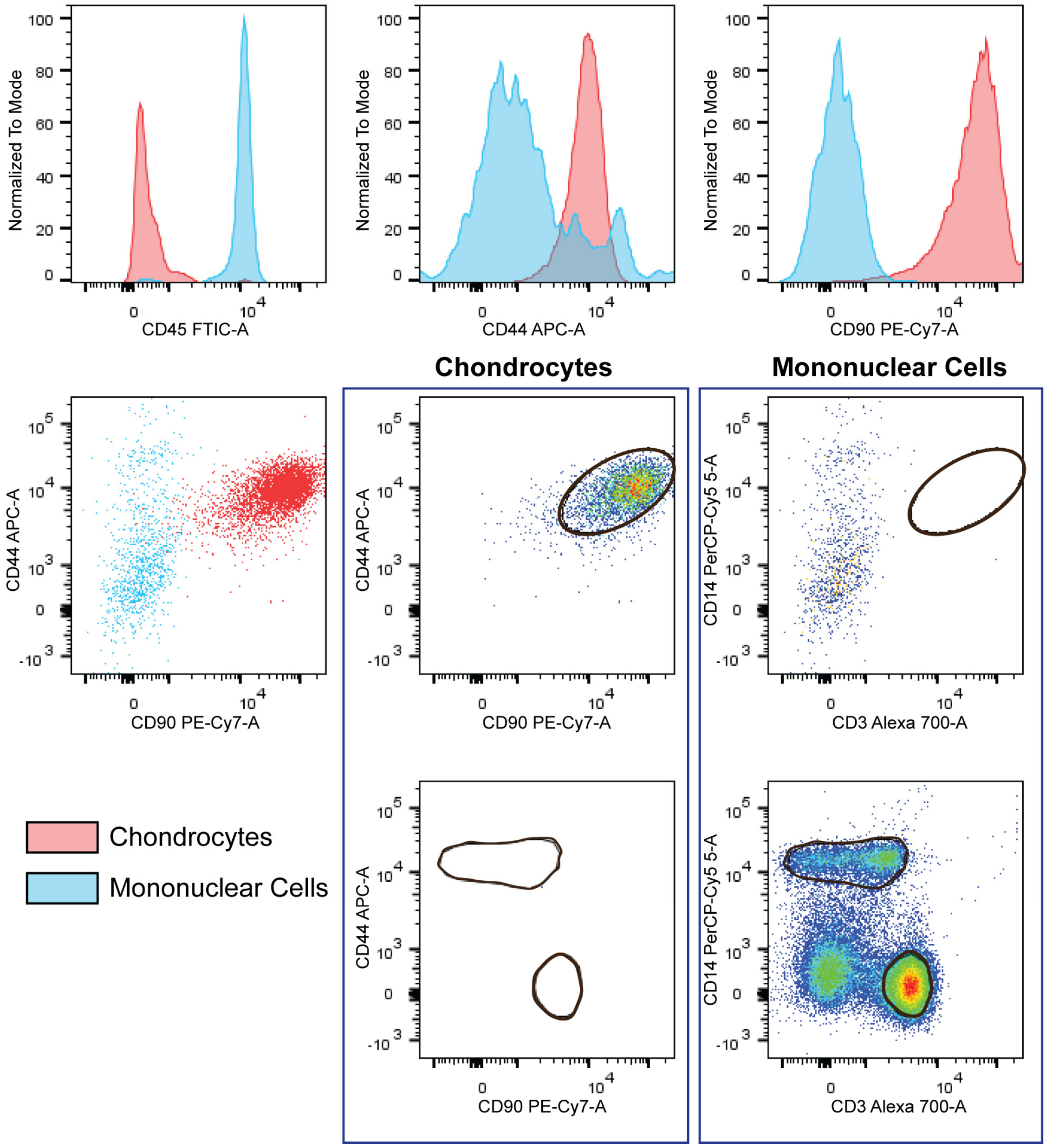

Supplementary Figure 2 Flow cytometry analysis for immune surface marker on polydactily chondrocytes. Polydactyly chondrocytes were stained with antibodies against immune-related surface markers, including CD45, CD14 and CD3. Mononuclear cells were used as immune cells controls. Polydactyly chondrocytes were also assessed for the chondrocytic markers CD44 and CD90.

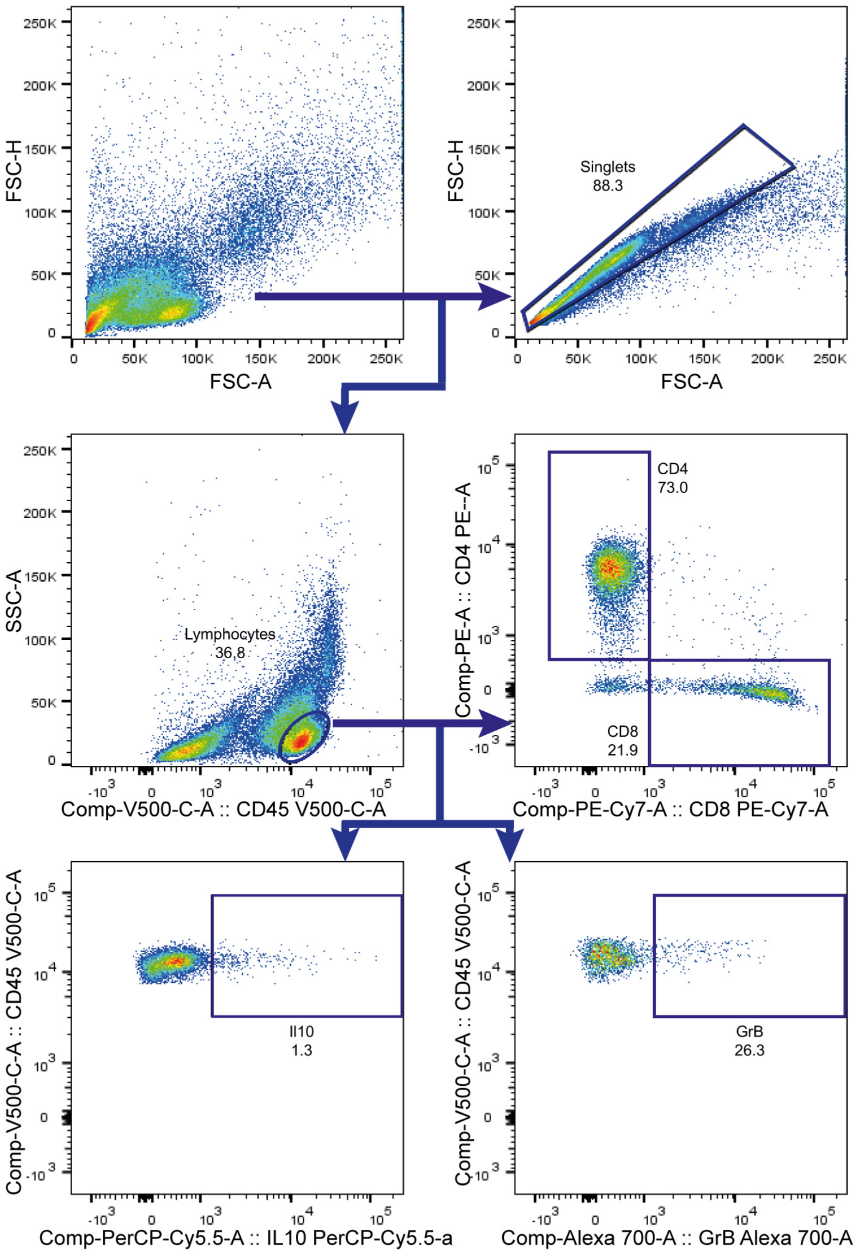

Supplementary Figure 3 Gating strategy for flow cytometry analysis of lymphocytes. The lineage cocktail used to measure T lymphocytes expressing IFNG, included, CD8, CD4, CD45, IL10, and GrB.

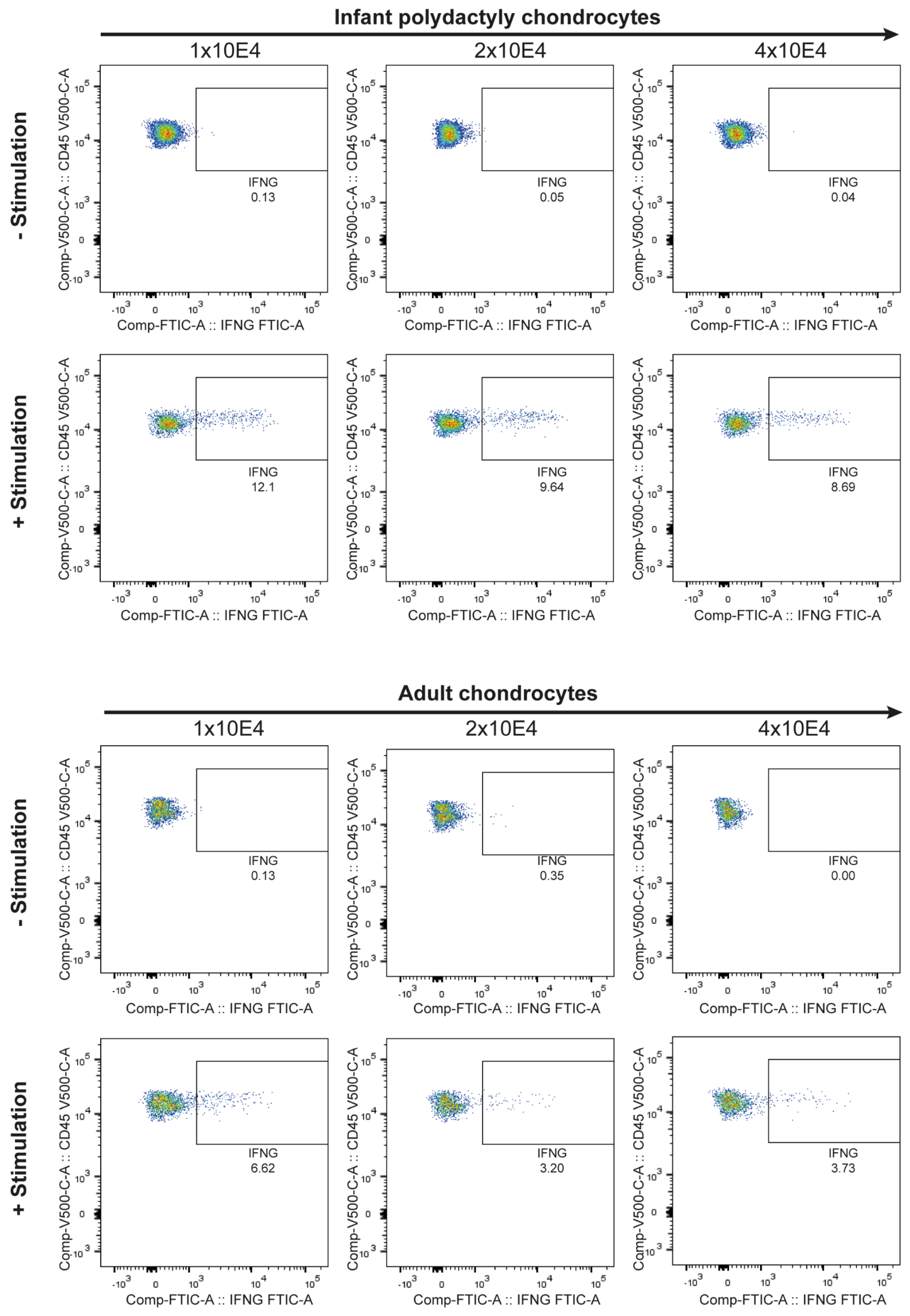

Supplementary Figure 4 CD8+ T-cell stimulation assay. (A) Infant polydactyly chondrocytes and (B) adult chondrocytes seeding gradient effect on T-cell stimulation assessed by IFNG production.

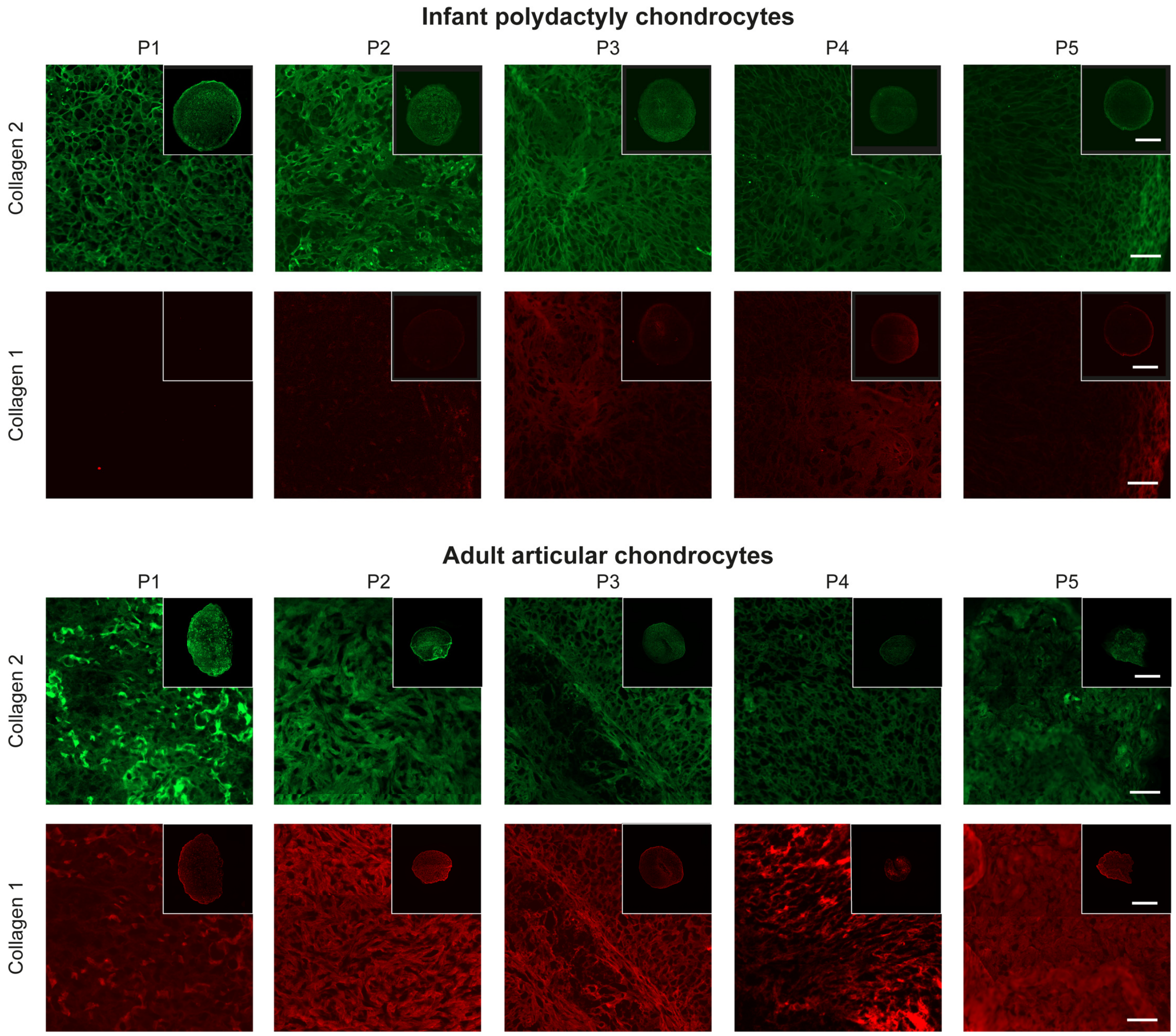

Supplementary Figure 5 Chondrogenic re-differentiation of infant polydactyly chondrocytes and adult articular chondrocytes in centrifuged pellet culture (Additional replicates). Collagen 2 and collagen 1 staining of centrifuged pellets made with 250’000 cells/pellet after 3 weeks of *in vitro* culture in chondrogenic media. Scale bar: 100 μm, scale bar insert: 500 μm.

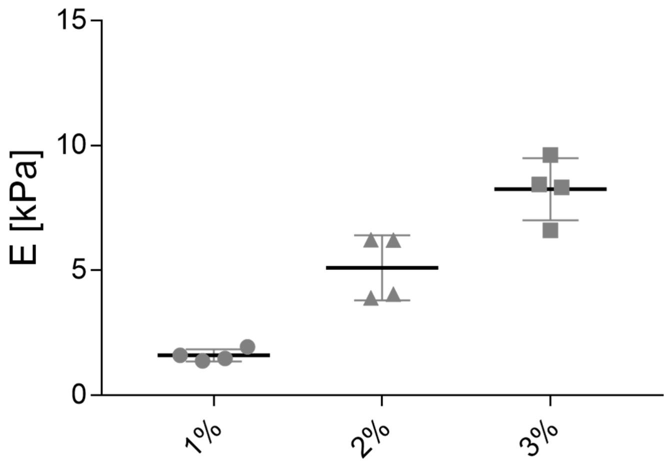

Supplementary Figure 6 Compressive modulus E of acellular 1, 2 and 3% w/v hydrogels.

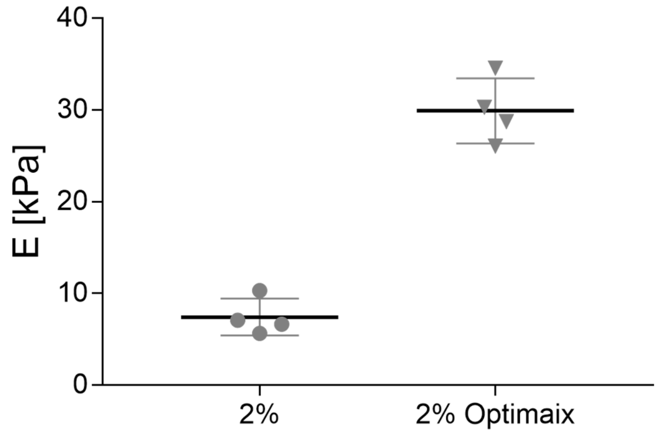

Supplementary Figure 7 Compressive modulus E of acellular 2% w/v HA-TG hydrogels with and without Optimaix scaffold.

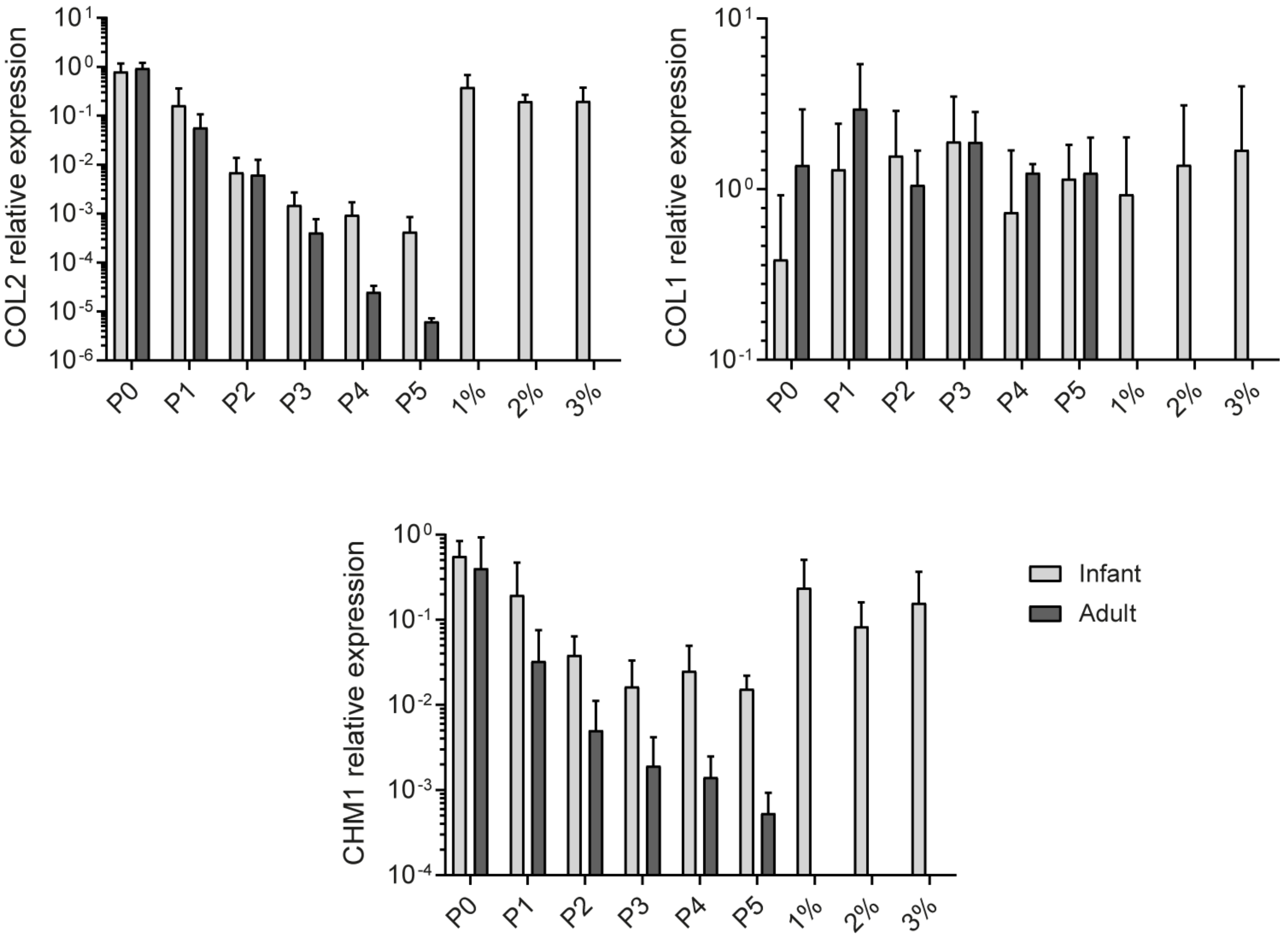

Supplementary Figure 8 Collagen 2, collagen 1 and chondromodulin 1 gene expression after sequential passaging and after re-differentiation of P3 infant chondrocytes in 1, 2 and 3% HA-TG hydrogels. Gene expression was normalized against the reference gene RPL13a with one infant chondrocyte donor at passage 0 chosen as the calibrator sample.
